# Redefining the Safe Donor Area in Follicular Unit Excision: Toward Dynamic, Patient‐Specific Zones

**DOI:** 10.1111/jocd.71250

**Published:** 2026-10-16

**Authors:** Sanusi Umar, Aditya K. Gupta, Vasiliki Economopoulos, Mesbah Talukder

**Affiliations:** ^1^ Dermatology Division, Department of Medicine University of California Los Angeles California USA; ^2^ Division of Dermatology Harbor‐UCLA Medical Center Torrance California USA; ^3^ Dr. U Hair and Skin Clinic Manhattan Beach California USA; ^4^ Mediprobe Research Inc. London Ontario Canada; ^5^ Division of Dermatology, Temerty Faculty of Medicine University of Toronto Toronto Ontario Canada; ^6^ School of Pharmacy BRAC University Dhaka Bangladesh

**Keywords:** donor miniaturization, follicular unit excision, hair transplantation, nape and periauricular hair, retrograde alopecia, safe donor area

## Abstract

**Background:**

Classical definitions of the safe donor area (SDA) identify a largely fixed occipital and parietal donor band presumed to remain permanent through a patient's lifetime. As a result of higher demands of extracting ever‐greater graft numbers using follicular unit excision (FUE), clinical situations have been highlighted where the purely anatomic, one‐size‐fits‐all SDA template is unreliable.

**Aim:**

Review the evolution of SDA concepts, summarize emerging evidence that qualifies static models, outline practical patient‐specific approaches to defining a conservative core SDA and, when appropriate, cautiously extending donor zones.

**Methods:**

We critically reviewed foundational SDA descriptions, observational and cohort studies assessing occipital donor variability and miniaturization, and practical tools to determine the donor zone, including the shave test, ratio‐based permanent zone estimation, and occipital fringe grading.

**Results:**

There is substantial variation in the occipital fringe position and donor miniaturization, with diffuse and retrograde thinning sometimes extending into areas traditionally considered safe. The nape and periauricular (NPA) areas in select patients can produce durable grafts resulting in natural appearing hairlines over midterm follow up. Subsequent ratio‐based and grading‐based approaches independently support patient‐specific inferior and superior borders, and reinforce that a single geometric SDA template is unlikely to apply in all patients.

**Conclusions:**

The SDA is a dynamic construct with a conservative core zone likely to remain permanent in most men, plus optional peripheral zones that may be incorporated only after careful assessment of occipital fringe position, miniaturization, and shave test findings, particularly along the inferior border.

## 1 Introduction

The safe donor area (SDA) identifies scalp regions where follicles remain resistant to androgenetic miniaturization over a patient's lifetime, so transplanted hair maintains long‐term coverage [1, 2, 3, 4, 5]. Classical SDA concepts arose when donor harvesting was confined to a narrow strip. These templates are conservative and remain useful for surgical planning [2, 6]. Contemporary evidence prompts reexamination of a fixed, landmark‐based SDA view. Diffuse and retrograde thinning within the occipital fringe is recognized in Asian/Indian datasets [7]. The need for finer caliber hair for hairlines, temples, brows, and lashes has increased interest in nape and periauricular (NPA) hair, historically avoided because of permanence concerns [8]. Some patients show strip scars that later lie in thinning zones as donor miniaturization progresses over time (Figure 1).

**FIGURE 1:**
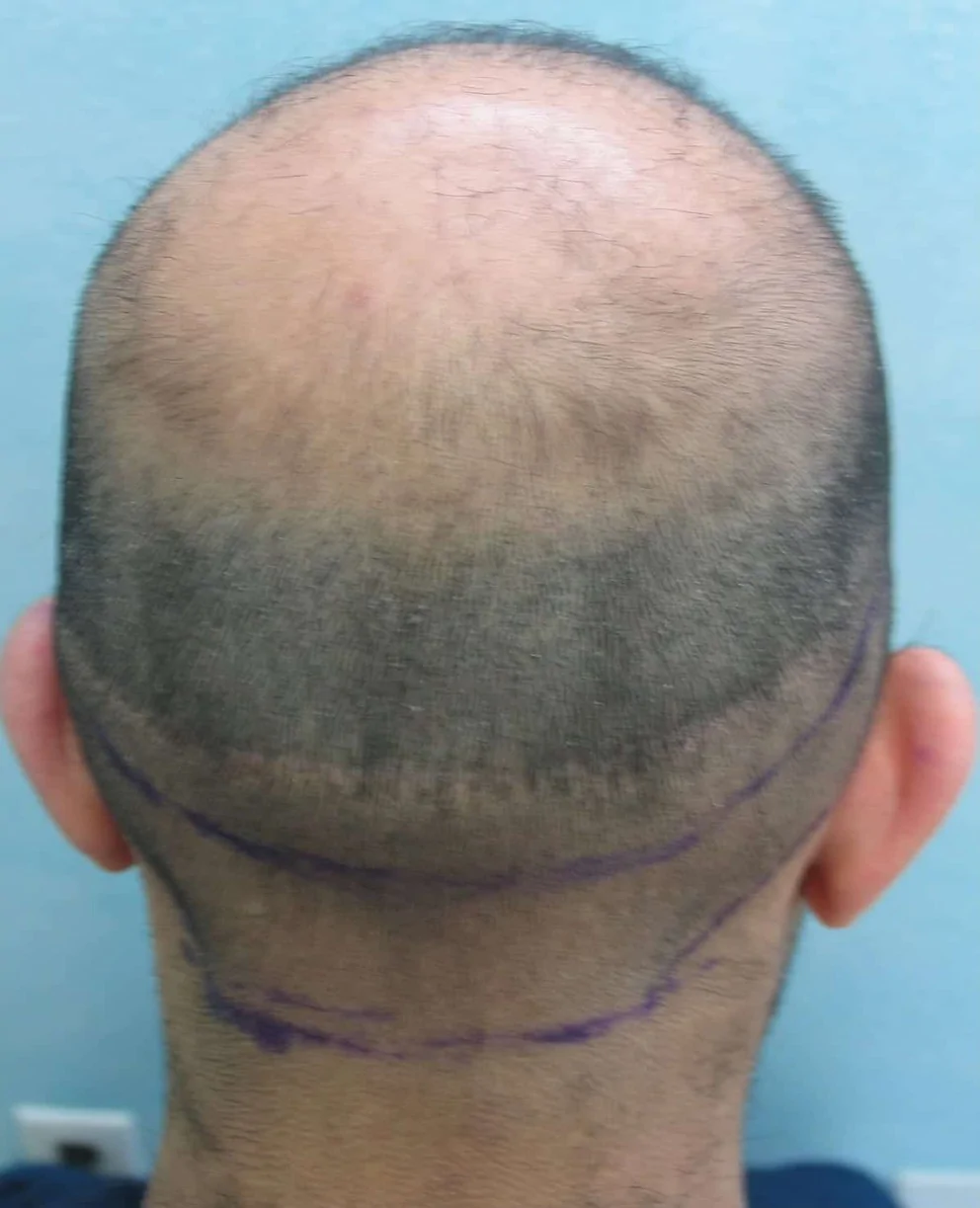
Example illustrating potential limitations of static, fixed anatomic safe donor area landmarks. This 35‐year‐old man underwent strip harvesting based on standard preoperative donor boundaries. Several years later, a shaved scalp evaluation demonstrated that the previously designated inferior donor zone now lies within an area of retrograde thinning. Such encroachment might have been recognized preoperatively with a shaved scalp assessment that visualizes the inferior donor transition (Shave test negative: Unstable donor area).

In advanced alopecia, donor supply can be limiting; objective, patient‐specific assessment may identify stable hair beyond rigid templates, allowing cautiously expanded harvesting that can add clinically meaningful graft numbers.

This review traces the SDA journey from fixed templates to dynamic, patient‐specific concepts, highlighting tools that define individualized borders, including the scalp shave test first described in 2015 and ratio and grading methods that support a flexible approach.

## 2 Methods

This narrative review was developed through a targeted literature search of PubMed/MEDLINE and Google Scholar from database inception to February 2026. Search terms included combinations of SDA, follicular unit excision, occipital donor, donor miniaturization, retrograde alopecia, nape, periauricular, and parietal whorl. We included peer‐reviewed clinical studies and cohort or cross‐sectional donor profiling studies addressing donor zone stability or methods for individualizing SDA borders, along with seminal foundational descriptions of the SDA.

## 3 Historical Concepts of the SDA

### 3.1 Donor Dominance and Early SDA Thinking

Following reports of Orentreich's principle of donor dominance, occipital hair was selected as a primary source of grafts due to androgen resistance. Early SDA definitions mapped a universal permanent zone based on cross sectional observations in older men [1, 2].

These population‐based templates have supported decades of successful practice. However, the assumptions underlying a static permanent zone are less reliable when diffuse harvesting is performed using the Follicular Excision Technique (FUE) widely across the back and sides of the scalp.

### 3.2 Unger's SDA

In Unger's 1994 landmark work examining 328 men aged 65 years or older, he described an occipital and parietal band in which at least 10 hairs were present within a 4 mm circle and in which fewer than 20% progressed beyond Norwood Class VII [2].

Unger translated these data into a reflected parallelogram shaped SDA with an occipital midline height of approximately 7 cm, bounded anterosuperiorly by a line from the tragus and superiorly by a line approximately 2 cm above the superior helix [2, 6].

This model is widely used because it is practical and intentionally conservative. In modern practice, it is often applied as a planning template that attempts to avoid harvesting from zones that might later become androgen sensitive in men with advanced pattern progression [2, 6].

### 3.3 Alt, Cole, and Bernstein and Rassman

Alt's approach refined donor geometry by defining an anterior edge at the external auditory canal and a superior border approximately 2 cm below a horizontal line from the superior helical rim, again treating the occipital donor band as broadly fixed [9, 10].

Cole [11] described a calculated donor template for FUE, including a 203 cm^2^ safe area subdivided into zones with differing densities and characteristics.

Bernstein and Rassman [12] estimated the classical donor region as roughly 25% of total scalp area, mainly in the occipital and parietal regions, and suggested that approximately half this area could be harvested over time without visible thinning.

By design, however, these authors provide population averaged templates rather than borders tailored to a specific patient's occipital fringe position and miniaturization pattern [2, 6, 7].

## 4 Fixed Anatomic SDA Templates in the FUE Era

With FUE, follicular units may need to be harvested from broader regions of the back and sides of the head. This flexibility, combined with interindividual variation, has made it easier to recognize clinical situations in which a strictly static SDA definition may be imprecise [7, 13].

### 4.1 Inter Individual Variation and Population Differences

Classical SDA definitions were derived from population data and not intended to be patient specific. Because occipital fringe position and donor thinning patterns vary among populations, diagrams derived from one cohort may not reflect donor patterns in others (Figure 1) [2, 7, 14].

Reports of individualized methods by Kumaresan and Deepa, Punia and colleagues, and Park and colleagues are summarized in Table 1. Collectively, these studies support a practical point: SDA templates are useful starting references, but individualization is advisable, especially near the inferior and superior borders [7, 14, 15].

**TABLE 1:** Overview of approaches used to define or refine the safe donor area in modern practice.

Approach	What it provides	Strengths	Key limitations and cautions	Typical use case
Unger template	Occipital and parietal donor band based on older male cohort and density criteria	Conservative and widely taught starting point	Population averaged; may not reflect individual fringe variability or donor miniaturization	Initial mapping of core SDA, especially for strip planning
Alt and related geometric templates	Fixed distances from ear landmarks to define donor borders	Simple and reproducible	Does not directly account for patient specific miniaturization or retrograde patterns	Quick visual estimation; training and orientation
Cole calculated donor approach	Template based zoning that accounts for differing densities across donor subregions	Encourages density aware harvesting strategy	Still template based; requires adaptation to individual miniaturization	FUE planning with attention to density variation
Occipital fringe grading	Grades inferior fringe position and profiles donor thinning patterns in populations	Highlights frequency of patterns that do not fit classical templates	Cross sectional; grading does not by itself predict individual future progression	Risk stratification; prompts conservative planning in unfavorable grades
Ratio method	Quantitative estimate of vertical donor extent using patient specific anatomic ratio and miniaturization point	Provides a numeric framework; reinforces a buffer below miniaturization line	Short follow up; requires accurate identification of transition point	Patient specific inferior border estimation, particularly in Indian men
Umar's Shaved scalp shave test	Visual assessment of caliber and density gradient to identify sharp demarcation or smooth transition at inferior and lateral borders	Operational bedside tool; directly visualizes retrograde patterns	Observational evidence; operator dependent; needs longer term multi center validation	Selecting candidates for conditional NPA harvesting and refining inferior border
Trichoscopy	Objective assessment of follicular unit density, shaft diameter variability, and miniaturization	Improves detection of diffuse donor thinning and diffuse unpatterned alopecia	Requires expertise and standardized measurement; does not replace clinical judgment	Screening donor suitability and informing safe excision planning

### 4.2 Miniaturization Within the Putative Permanent Zone

Diffuse and retrograde thinning can occur within traditionally safe areas with implications for both strip and FUE procedures. Donor thinning can increase scar visibility and transplantation of miniaturized follicles may reduce long‐term recipient coverage (Figure 1) [7, 16].

Kumaresan and Deepa [7] defined diffuse thinning trichoscopically as a follicular unit density of < 30 follicular units/cm^2^ across sampled occipital, parietal, and temporal points, while retrograde patterns were identified clinically and supported by trichoscopic variability in hair shaft diameter.

Punia and colleagues [14] emphasized the variability of the inferior miniaturization line and suggested leaving a small buffer zone below this line unharvested to avoid dihydrotestosterone sensitive follicles.

### 4.3 Contemporary Clinical Pressures at SDA Borders

Higher expectations for density, large area recipient coverage, growth of high volume FUE extraction sessions, unregulated FUE practice, have all increased the likelihood of harvesting beyond classical ‘safe’ borders. This can contribute to visible donor thinning [13, 17].

The desire for softer, more natural hairlines has renewed interest in finer caliber hair from the NPA zones, areas that most classical diagrams exclude [8].

These realities raise a practical question: can selected hair from outside classical SDA maps be incorporated safely, and if so, how should safe be defined in a way that respects both traditional anatomic landmarks and patient‐specific patterns?

## 5 Dynamic SDA Concepts and Emerging Tools

Several methods have been proposed to individualize the SDA by incorporating scalp specific findings rather than relying solely on fixed distances [7, 8].

### 5.1 Shaved Scalp Shave Test and Use of NPA Hair

In 2015, Umar reported an observational FUE series where NPA hair was used to construct softer hairlines and temples in 128 patients, with postoperative follow up collected using patient questionnaires. Before surgery, each patient underwent a shaved scalp assessment, commonly referred to as the shave test, in which the occipital, temporal, and NPA regions were clipped short and the pattern of hair density and caliber transition was visually assessed [8].

Patients considered candidates for NPA harvesting demonstrated a smooth, gradual decline in hair caliber and density from the mid‐occipital donor region into the NPA areas without a sharp line of demarcation (Shave test positive: stable donor area) (Figure 2). Patients considered noncandidates showed a sudden step‐down in caliber or density with a distinct inferior border and, in some cases, visible retrograde thinning extending into the occipital fringe (Shave test negative: unstable donor area) (Figure 3) [8].

**FIGURE 2:**
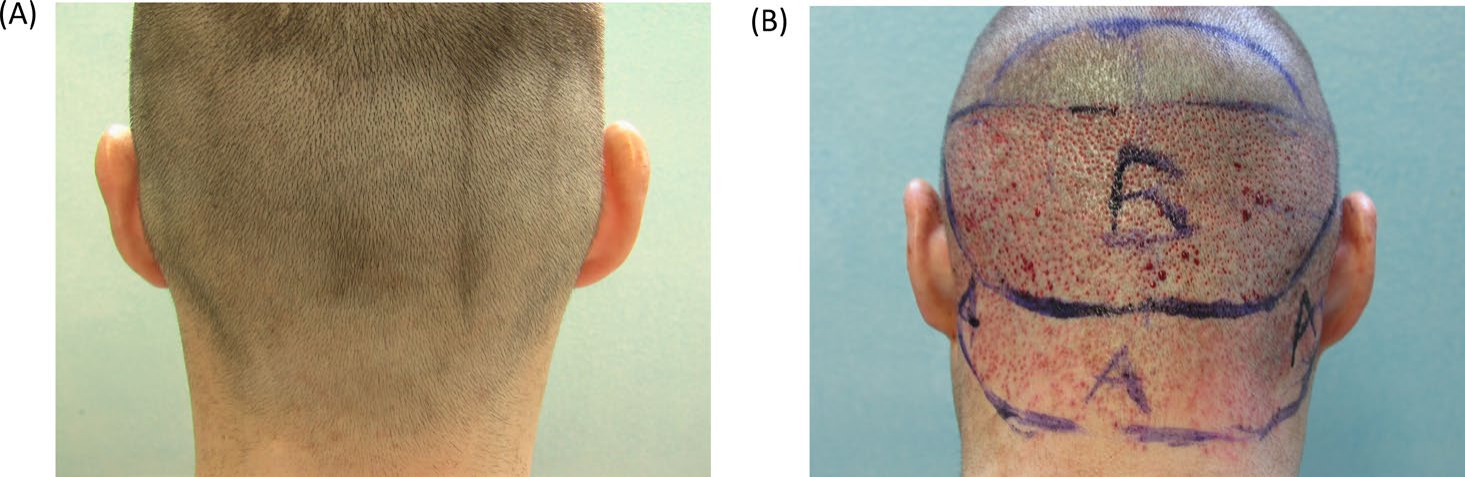
A shave test–positive: Stable donor area man, ages 32. After shaving (A) and after FUE extraction (B), demonstrating a smooth, gradual transition in hair caliber and density from the mid‐occipital safe donor zone into the nape, periauricular, and temple regions. The shaved‐scalp pattern (Shave test positive) suggests that these inferior and lateral zones behave as part of the patient‐specific permanent donor area. In static anatomic SDA diagrams (Zone 1), these extended regions (Zone 2) are typically excluded, illustrating how dynamic, pattern‐based assessment can refine traditional templates. Reproduced with permission from ref. [8].

**FIGURE 3:**
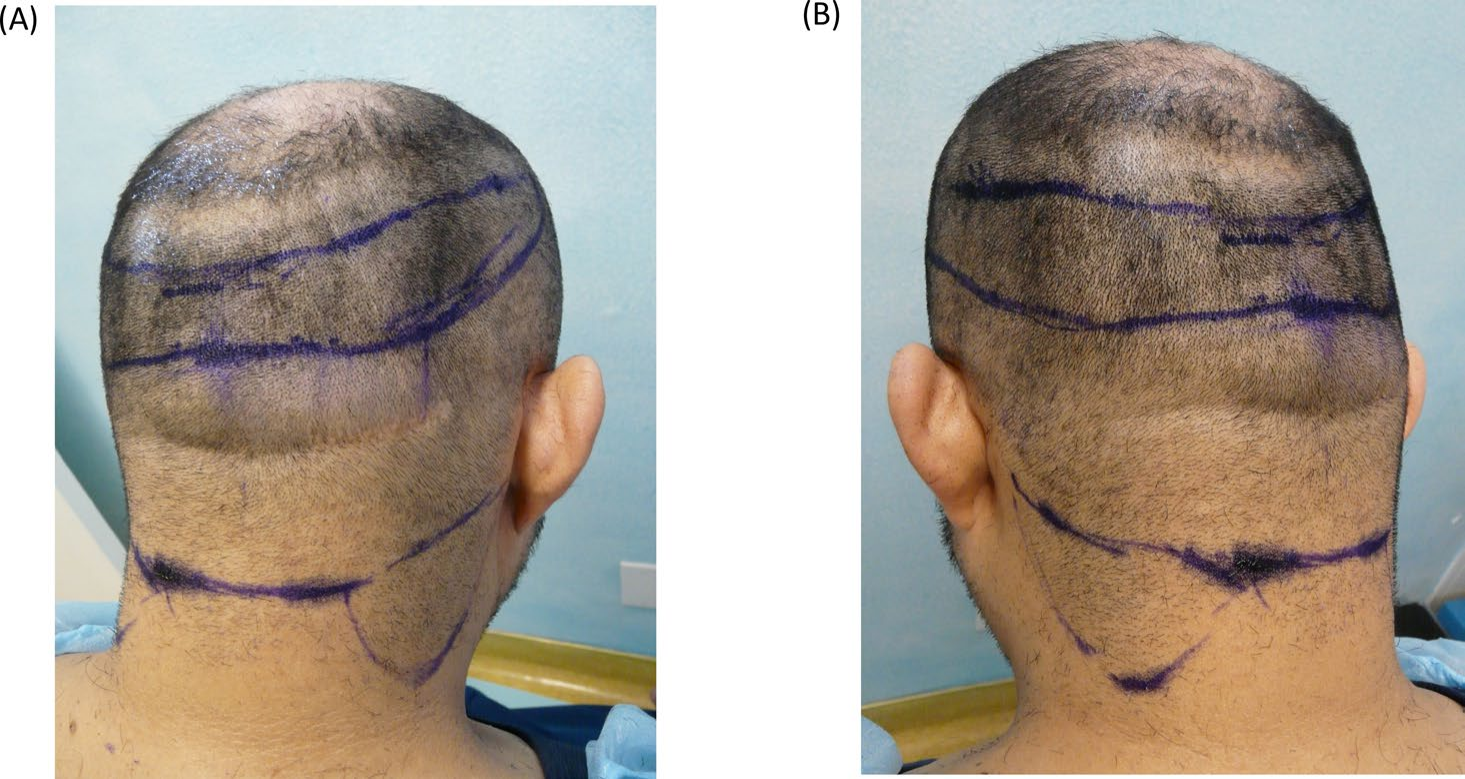
Clinical examples of shave‐test–negative: Unstable donor area patients with retrograde thinning. (A, B) A man in his mid‐30s with a distinct line of demarcation between the mid occipital donor region and the inferior retrograde zone characterized by a precipitous fall in hair caliber and density. The visible transition delineates an inferior donor boundary and identifies zones that are best avoided for scalp donor harvesting.

In this cohort, 91 of 128 patients (71%) responded to the first postoperative questionnaire 25.5 months on average after the procedure. Respondents reported complete postoperative recipient coverage at 9.6 months and reported a mean overall satisfaction score of 8.3 on a 10‐point scale [8].

A second questionnaire was completed by 33 respondents at an average of 4.6 years after surgery, with a mean satisfaction score of 8.5. Only five new instances of partial graft loss were reported in the second survey. Based on recall data from eight patients, mean recipient area hair loss was estimated at 24% [8].

Importantly, in some patients with graft loss, this involved transplants from both the NPA (nape and pre‐auricular area) and the conventional donor region. This supports the view that donor permanence is not absolute, which is consistent with miniaturization extending into traditional donor zones in AGA, although less than in the primary balding areas [18, 19, 20].

In that series, NPA (nape and pre‐auricular area) derived grafts were primarily placed in transitional zones such as the leading edge of the hairline and temple points to minimize conspicuous cosmetic impact should limited future NPA miniaturization occur. Any changes that occur at the leading edge can resemble natural hairline maturation rather than an abrupt defect [8, 17].

In anecdotal long‐term (≥ 10 years) follow‐up, transplanted NPA hairlines appeared preserved in shave‐test–positive patients: stable donor area despite continued recession of the native frontal hairline (Figure 4).

**FIGURE 4:**
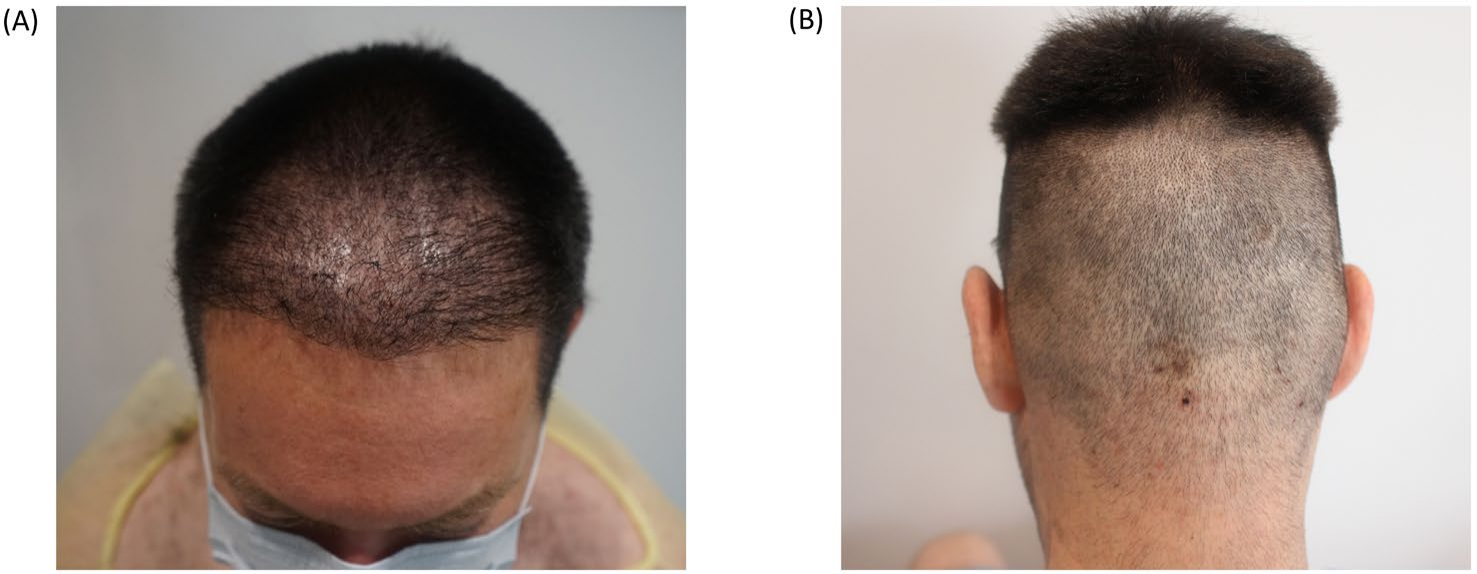
This is the patient in Figure 2. He was shave test positive: Stable donor area at pre‐op. At 14 years post‐FUE procedure the top frontal view shows significant progression of recession of his native hairline and a stable transplanted hairline, including vanguard NPA (nape and pre‐auricular area) hair (A), the donor area demonstrates NPA zone stability (B).

The shave test is noteworthy because it represented an early published bedside approach that allowed for patient specific assessment of the inferior donor border; however, large scale trials are needed to validate this approach. Regardless this approach demonstrates that donor borders are most safely defined around the patient's actual fringe pattern and miniaturization profile [7, 8, 14].

Additional individualized methods that can be considered and used in conjunction with the shave test are the ratio method for permanent zone estimation by Punia and colleagues, and the occipital donor grading and profiling system described by Kumaresan and Deepa, which are summarized in Table 1.

### 5.2 Other Adjunctive Approaches

Adjunctive approaches that can complement dynamic SDA planning include population specific anatomic markers such as parietal whorl position, as described in Korean men [15], and systematic donor site assessment using trichoscopy [20, 21]. Trichoscopy can assist in excluding diffuse unpatterned alopecia, identifying diffuse donor thinning, and estimating follicular unit density and hair shaft diameter and inform both donor zone selection and safe excision limits for FUE [20].

Practical guidance on maintaining esthetic donor density, with FUE excision planning, has also been described, reinforcing that donor capacity depends not only on donor area size but also on baseline density, hair shaft diameter, and excision pattern used during harvesting [13].

## 6 Integrating the Evidence: A Practical Dynamic SDA Framework

### 6.1 Step 1: Establish a Conservative Core SDA

Classical templates (Unger, Alt, Cole, Bernstein and Rassman) can be used to map an initial core SDA within the occipital and parietal scalp that can be treated as a starting point [2, 9, 11, 12]. Trichoscopic assessment can then be used to screen for diffuse unpatterned alopecia, diffuse thinning, or retrograde thinning patterns that might reduce donor stability [7, 20, 21]. Occipital fringe grading can help identify patients whose fringe position or thinning pattern suggests a narrower donor zone and a need for conservative harvesting even within classical boundaries [7].

### 6.2 Step 2: Refine Superior and Inferior Borders With Patient Specific Tools

For the superior border, expected vertex expansion based on current pattern, age, and family history can be combined with population specific markers such as parietal whorl position when relevant [15].

For the inferior border, combine visual and trichoscopic identification of the miniaturization transition with a shaved scalp assessment. The shave test focuses on the density and caliber gradient from the mid‐occipital area into the NPA fringe and flags abrupt step offs that suggest retrograde thinning [8, 14, 20].

Patients whose shaved scalp phenotype shows a smooth gradient without a clear demarcation line are more likely to have a stable inferior fringe, whereas patients with a sharp transition or established retrograde thinning are better managed with a narrowed inferior donor boundary [7, 8].

### 6.3 Step 3: Define Optional Extended Zones

Only after a conservative core SDA is defined should optional peripheral zones be considered. These zones should be viewed as conditional and indication specific rather than universally permanent [8, 17].

NPA hair: In shave test positive patients, a limited band of NPA hair may be considered as a conditional extended donor source for indications such as soft hairlines and temples. The published 2015 series suggests acceptable midterm durability when patients are carefully selected and when NPA grafts are concentrated in transition zones [8].

Anterior parietal border zones: In patients with stable fringe patterns (e.g., Favorable shave test) and reassuring trichoscopy, limited harvesting from anterior parietal regions may be considered, but long‐term outcome data remain limited and conservative planning is recommended [7, 13].

Patients with early onset androgenetic alopecia, strong family histories of extensive pattern loss or retrograde thinning or negative shave test or evidence of donor miniaturization in intermediate zones are generally poor candidates for extended scalp donor harvesting. For these patients, a smaller scalp donor zone and, when acceptable, greater reliance on beard or body hair may be safer [4].

Table 1 summarizes commonly used classic templates and patient‐specific tools that can be combined to operationalize a dynamic SDA approach.

## 7 Patient Selection, Counseling, and Medical Therapy

Regardless of the specific tool used to estimate donor borders, several shared clinical principles emerge from the available literature and expert guidance [7, 13, 14, 16]. (1) History and family pattern: Early onset androgenetic alopecia, rapid progression, and strong family history of extensive pattern loss or retrograde thinning support a conservative approach and argue against extended donor harvesting [7, 8]. (2) Scalp and donor examination: Evidence of retrograde thinning, diffuse thinning, or miniaturization in the occipital fringe, nape, periauricular region, or anterior parietal zones on shaved scalp inspection or trichoscopy should shift borders and reduce harvestable areas [7, 8, 20, 21]. (3) Age at surgery and staging: Younger patients are more likely to progress. Reassessment over time and staged procedures can be safer than a single large session, particularly when extended zones are considered. (4) Medical therapy: Long‐term finasteride or dutasteride and topical or oral minoxidil may help stabilize androgenetic alopecia, but some patients are unwilling or intolerant [22]. In such cases, the threshold for using extended zones should be higher (confine extraction predominantly to within traditional SDA). (5) Consent and expectation management: No method can guarantee lifelong graft permanence. Patients should understand that any extended zone carries a nonzero risk of future miniaturization  [7, 8, 13].

## 8 Future Directions

Strategies for defining and utilizing the SDA continue to evolve. There are several priority developments that would improve the current evidence base: (1) Prospective, multicenter longitudinal studies combining shave test phenotypes, ratio‐based calculations, and occipital fringe grading with objective trichoscopic and standardized photographic follow up for 10 years or longer [7, 8, 14]. (2) Standardized documentation of NPA (nape and pre‐auricular area) and other extended zone graft survival, including studies that stratify outcomes by recipient indication (hairline, temples, eyebrows) and by patient risk profile [8]. (3) Integration of imaging and automated density mapping to improve reproducibility of donor assessment while preserving clinical judgment.

## 9 Conclusions

The SDA concept has evolved from static strip‐based templates to a more nuanced dynamic construct informed by the patient's occipital fringe position and miniaturization pattern [2, 7].

Classical diagrams remain valuable as conservative starting points. However, larger population datasets show that a meaningful proportion of patients do not fit traditional templates and that donor thinning patterns can extend into zones once assumed to be uniformly safe [7, 14, 16].

Practical tools such as shaved scalp assessment (shave test), ratio‐based donor estimation, occipital fringe grading, and trichoscopy all embody the same principle: define donor borders around what the patient's scalp demonstrates rather than relying on a fixed diagram alone [7, 8, 14, 20].

Within this framework, NPA (nape and pre‐auricular area) and other peripheral zones may be considered conditional donor sources in carefully selected patients and for specific indications, particularly for soft hairline transitions [8].

## Author Contributions

Conception of the manuscript was done by Sanusi Umar. The manuscript was drafted by Sanusi Umar, Aditya K. Gupta, Vasiliki Economopoulos and Mesbah Talukder. The manuscript was substantively edited and revised by Aditya K. Gupta, Sanusi Umar and Mesbah Talukder.

## Funding

The authors have nothing to report.

## Consent

The authors confirm that written informed consent for publication has been obtained for all clinical photographs included in this manuscript.

## Conflicts of Interest

The authors declare no conflicts of interest.

## Data Availability

No new data has been produced.
